# Levothyroxine absorption test followed by directly observed treatment on an outpatient basis to address long-term high thyrotropin levels in a hypothyroid patient: a case report

**DOI:** 10.1186/s13256-023-03760-0

**Published:** 2023-01-25

**Authors:** Zareen Kiran, Khalilullah Shabbir Shaikh, Nazish Fatima, Namra Tariq, Akhtar Ali Baloch

**Affiliations:** National Institute of Diabetes and Endocrinology, Dow University of Health Sciences, Ojha Campus, SUPARCO Road, KDA Scheme-33, Karachi, 74200 Pakistan

**Keywords:** Levothyroxine, Hypothyroidism, Levothyroxine absorption test, Compliance, Pseudo-malabsorption

## Abstract

**Background:**

For the treatment of hypothyroidism, patients are usually placed on lifelong levothyroxine replacement therapy. Achieving clinical and biochemical euthyroid status is sometimes difficult because of several factors, including poor compliance or poor absorption of levothyroxine in the gut mucosa.

**Case presentation:**

We present the case of a 45-year-old South Asian female with hypothyroidism on lifelong levothyroxine replacement. However, on her multiple follow-up visits over the past 2 years, it was noted that her thyrotropin level was never controlled and was not in range. A thorough history was unyielding in terms of compliance regarding levothyroxine medication and use of over-the-counter pills or mineral supplements that may interfere with the absorption of levothyroxine. Hence, we performed levothyroxine absorption test to rule out any malabsorption as well as document pseudo-malabsorption favoring drug nonadherence. Levothyroxine absorption test resulted in more than 56% absorption of levothyroxine; therefore, the patient was put on weekly directly observed treatment strategy resulting in normalization of thyrotropin levels on successive follow-ups.

**Conclusion:**

Directly observed treatment to replace levothyroxine once a week can be used as an alternative by health care professionals in patients in whom compliance to daily levothyroxine is the hidden cause of persistently raised thyrotropin. This strategy can reduce undue health burden on the patient and improve adherence to self-medication under confident supervision of the treating physician.

## Background

Levothyroxine (LT4) is considered as the drug of choice in treating patients with hypothyroidism, because of its efficacy, long-term safety, low cost, good absorption rate, and greater half-life [1]. Levothyroxine is usually taken in fasting state because it needs physiological gastric pH for its maximum absorption. The percentages of thyroxine (T4) absorbed had been estimated between 17% and 93% of the ingested dose with an overall value of 60–80% [2]. Numerous conditions, either physiological, pathologic, nutritional, or pharmacologic, may reduce the absorption of levothyroxine. Gastritis, gastroparesis, drugs including multivitamins, proton pump inhibitors etc., bariatric surgery, and intake of certain foods such as coffee, milk, and grapefruit juice may decrease the levothyroxine absorption from intestine [2]. Levothyroxine is usually started at a dose of 1.6 µg/kg/day. The failure to achieve euthyroid status biochemically after taking levothyroxine can be due to multiple reasons such as poor compliance to medication, taking insufficient dose, taking levothyroxine with food, malabsorption due to gastrointestinal disease such as celiac disease, and drug interaction with thyroxine [3]. Patients who have poor compliance to daily thyroxine therapy may benefit from once-weekly oral or intravenous levothyroxine [4].

We report the case of a hypothyroid female patient, with persistently elevated thyrotropin (TSH) despite receiving long-term levothyroxine therapy. Weekly levothyroxine treatment resolved the question between true malabsorption and pseudo-malabsorption through directly observed treatment strategy (DOTS) in this patient. This is the first case to be reported from Pakistan.

## Case discussion

**Fig. 1:**
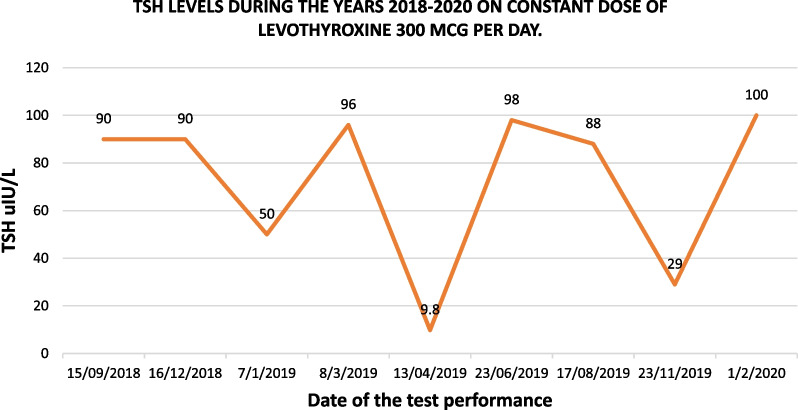
Serum thyrotropin trend on levothyroxine 300 µg per day dose over the past 3 years

A 45-year-old South Asian lady was referred to our institute, because of persistent elevation of TSH even after taking increasing dose of levothyroxine for 3 years. She was diagnosed with hypertension, rheumatoid arthritis, dyslipidemia, and hypothyroidism for 25 years. She was taking hydroxychloroquine, omeprazole, paracetamol, and diclofenac sodium in addition to levothyroxine 300 µg (six 50 µg tablets per day). She was recently started on hydroxychloroquine and azathioprine for a rheumatoid arthritis flare-up about 6 months ago. She had a hysterectomy 25 years ago, and she had no other significant surgical or medical history. Her family history was negative for thyroid or other autoimmune disorders. She lives in a joint family from a lower socioeconomic class. Her husband works as a shopkeeper and is the family’s sole breadwinner. When she presented to us, she complained of fatigue, dry skin, and constipation. She was stable on examination, with a pulse of 78 beats per minute, blood pressure of 128/79 mmHg, weight of 49 kg, and height of 150 cm [body mass index (BMI) 21.7 kg/m^2^]. Her thyroid gland was not enlarged, and she had no rheumatoid arthritis deformities. She had dry skin, particularly on her elbows, and had hair loss on both of her legs. Her ankle deep tendon reflexes were slowly releasing. Table 1 presents routine investigations done three years back, whereas Fig. 1 shows the TSH levels over the past 3 years and concomitant levothyroxine dose advised by her general physician. After thoroughly reviewing her history and examination, we reviewed her compliance with levothyroxine drug and dosage, and the use of concomitant medications was assessed. She was instructed to obtain levothyroxine from a reputable medical store, and she and her husband were counseled on the importance of lifelong need of hormone replacement therapy. After the first visit, her TSH dropped down to 9.8 µIU/L (Fig. 1), so her dose was readjusted. Her TSH returned to 98 µIU/L on follow-up, so her dose was increased once more. However, her TSH did not improve significantly. At this point, her hemoglobin was found to be 10.7 g/dl, and she was started on iron supplements at lunchtime. Additional testing was recommended to rule out the possibility of malabsorption syndromes. Her anti-tissue transglutaminase antibodies A and G (anti-TTG IgA and IgG) were negative, and her vitamin D levels were very low, but her vitamin B12 levels and serum albumin were normal. Serum iron levels were low. Vitamin D was adequately replaced, and she was advised to follow up. It was decided at this point to perform a levothyroxine absorption test to distinguish between pseudo and true malabsorption in this case.

**Table 1:** Routine laboratory investigations prior to attending National Institute of Diabetes and Endocrinology, Dow University of Health Sciences

Laboratory tests	18 April 2018	29 July 2018	18 April 2018	Normal values
Fasting blood sugar	130	90		< 100 mg/dl
Low-density lipoprotein cholesterol	259	152		< 100 mg/dl
Triglyceride	274	233		< 150 mg/dl
Total cholesterol	369	234		< 200 mg/dl
Hemoglobin A1c	6.3%			< 5.7%
Erythrocyte sedimentation rate		95	52	0–29 mm first hour
Anti-cyclic citrullinated peptide		55.9		< 5 U/ml

### The levothyroxine absorption test

Patient was asked to come in a state of overnight fasting (at least 8 hours). She arrived in our outpatient department (OPD) at 8:30 am, and blood samples were drawn at 09:25 am. She was then given 20 tablets of levothyroxine 50 µg [the brand available in our city was Thyroxine at the standard dose of 50 µg manufactured by GlaxoSmithKline (GSK) pharmaceutical company] according to the following protocol at 10:00 am:

Standard LT4 weekly dose:

- Age 18–65 years, BMI > 40 kg/m^2^: 1500 µg = 30 tablets.
- Age 18–65 years, BMI < 40 kg/m^2^: 1000 µg = 20 tablets.
- Age 65 years and older: 600 µg = 12 tablets.

She remained seated in the consultant’s OPD room for 1 hour, and then allowed to eat cookies. A second blood sample was drawn at 1:26 pm (Table 2).

**Table 2:** Levothyroxine absorption test

24 August 2020	Timings	Result
Free T4, pg/ml (0.85–1.76)	9:25 am	1.15
Total T4, ng/ml (5.5–11.0)	9:25 am	8.0
Thyroxine 50 µg (by GSK) 20 tablets given orally as DOTS (directly observed treatment strategy)		
Free T4, pg/ml (0.85–1.76)	1:26 pm	2.05
Total T4, ng/ml (5.5–11.0)	1:26 pm	13.9

### Interpretation of test

The percentage LT4 absorption is calculated using the following formula [5]:

o % Absorbed = [increment total T4 (TT4) µg/dL × 10/total administered LT4 µg] × Vd (L) × 100.
p Increment TT4: peak [TT4] – baseline [TT4].
q Vd (volume of distribution) = 0.442 × BMI (body mass index)

Normal absorption was considered to be greater than 60% [6, 7]. This means pseudo-malabsorption of LT4 due to intentional nonadherence. However, if it is less than 60%, it means true malabsorption. We entered patient’s data by applying the above formula as shown in the Box﻿ 1.

The results were suggestive of pseudo-malabsorption rather than true malabsorption. For further management, patient was asked to come every week with overnight fast for 5 weeks. On each visit, 20 tablets of levothyroxine of same brand were given and patient was asked to sit in the OPD for 1 hour under direct observation of the treating endocrinologist. Her TSH continued to be normal after several months of DOTS therapy as shown in Table 3.

**Table 3:** Comparison of TSH before and after directly observed treatment strategy

	9 August 2020	24 August 2020		1 October 2020
TSH, µIU/L (0.4–4.2)	41.73			0.73
Time		9:45 am	1:26 pm	
Free T4, pg/ml (0.85–1.76)		1.15	2.05	1.50
Total T4, ng/ml (5.5–11.0)		8.0	13.9	9.4
Dose of levothyroxine (50 µg)	3 tablets (Monday to Friday) and 2 tablets (Saturday and Sunday)	Thyroxine 50 µg 20 tablets given orally as DOTS		On 20 tablets per week

Box 1. Patient data and result

- Increment TT4: peak [TT4] – baseline [TT4] = 13.9 – 8.0 = 5.9
- Total administered dose = 1000 µg = 20 tablets
- Vd (volume of distribution) = 0.442 × BMI = 0.442 × 21.7 = 9.59
- where BMI was 21.7 kg/m^2^.
- So, after putting all variables in formula of % absorbed, our result was 56.58%.

## Follow-up

**Fig. 2:**
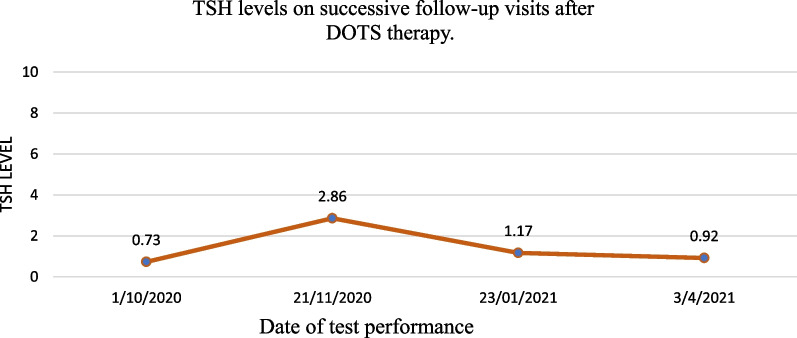
TSH levels on successive follow-up visits after DOTS therapy

The patient’s husband has been asked to follow DOTS protocol at home on weekly basis. This resulted in a continuous normal range of TSH on follow-up as shown in Fig. 2. Therefore, we concluded that her raised TSH was secondary to poor compliance to oral levothyroxine therapy.

## Discussion

More than 90% of hypothyroidism cases are caused by primary hypothyroidism. Because this illness is chronic, the majority of these patients will require levothyroxine therapy for the rest of their lives. Adherence to levothyroxine, on the other hand, gradually decreases over time. Early detection and proper counseling, as well as modifying the regimen as needed, are required to reverse the potential complications and prevent the life-threatening myxedema coma [8].

In our case, the patient had hypothyroidism for 20 years and was treated with levothyroxine. She was referred to the endocrine department due to her persistently elevated TSH despite taking levothyroxine on a daily basis. By responding to the following questions, the common causes of TSH elevation even after daily levothyroxine administration can be addressed. Firstly, the biggest challenge is to ensure adherence to levothyroxine medication, as in a majority of cases noncompliance to medication is the main reason behind failure to achieve euthyroid status [8]. Second, it is prudent to inquire about any over-the-counter medications that the patient may be taking concurrently and that may have interfered with levothyroxine absorption [2]. Notably these medications are multivitamins, iron and calcium supplements, rifampin, anti-epileptics, proton pump inhibitors, cholestyramine, imatanib, and sucralfate [8]. Third, thyroxine should be taken on an empty stomach for maximum absorption, and no other medication or food should be taken for at least 30–60 minutes after levothyroxine ingestion [2]. Fourth, investigations regarding malabsorption should be done to exclude any chance of levothyroxine malabsorption from gastrointestinal tract because levothyroxine is usually absorbed from small intestine especially jejunum and ileum. In this context, a levothyroxine absorption test can be performed to distinguish between those who have true malabsorption and those who have raised TSH due to medication nonadherence or pseudo-malabsorption. Because there is no gold standard method for the LT4 absorption test, different protocols have been advocated in the literature. The majority of centers use 4- and 6-hour sampling to determine the trend of total T4 and FT4. Some have also tested for rapid inhibition of TSH. When all published pseudo-malabsorption cases are considered, it has been stated that at least two to three times increase in the basal FT4 levels may exclude malabsorption [9]. Similarly, in certain medical conditions like nephrotic syndrome, large amounts of albumin are excreted, which may increase levothyroxine requirements due to binding of T4 to the excreted albumin. This creates a milieu of protein malnourishment that may be difficult to differentiate from malabsorption syndromes [10]. Finally, potential side effects of giving higher doses of thyroxine should be kept in mind, such as once-weekly dose, especially in the elderly and those with prior ischemic heart disease, as they may develop or precipitate tachyarrhythmia; therefore, DOTS therapy should not be used in this group of patients.

Our patient already had two autoimmune conditions; therefore, it was critical to consider celiac disease as one of the possible autoimmune absorptive diseases causing impaired levothyroxine absorption. Despite the fact that the celiac serology was negative, the question arose as to whether we should still refer the patient for a duodenal biopsy. We deferred this step because the history and laboratory workup revealed no significant malabsorption and our patient’s TSH remained in the normal range on follow-up.

## Conclusion

We took a methodical approach to our patient’s chronically elevated TSH. Laboratory investigations and levothyroxine absorption test both ruled out true malabsorption. Levothyroxine was started as a directly observed treatment strategy, first in front of the treating endocrinologist and then under the direct supervision of her husband at home. On weekly DOTS, the patient’s medication compliance was satisfactory, and she was not taking any additional drugs that would have affected levothyroxine absorption. Hence, we conclude that a trial of a single weekly bolus dosage of levothyroxine under the guidance of a health care professional or family member is a reasonable strategy to adopt for these patients. This is particularly helpful in countries like Pakistan, with limited health care resources, and where patients have different cultural myths related to several medical as well as endocrine diseases. Moreover, such simple test and trial of DOTS may help resolve the effect of polypharmacy and save the patient from unnecessary advanced investigations.

## Data Availability

The dataset supporting the findings of this study can be made available upon request to the first author, whose email is zareen.kiran@duhs.edu.pk, drzareenkiran@gmail.com.

## Abbreviations

LT4: Levothyroxine
T4: Thyroxine
TSH: Thyrotropin
DOTS: Directly observed treatment strategy
BMI: Body mass index
Anti-TTG IgA and IgG: Anti tissue transglutaminase antibodies A and G
OPD: Outpatient department
FT4: Free T4
GSK: GlaxoSmithKline
Vd: Volume of distribution
